# Teasing Children

**Published:** 1871-01

**Authors:** 


### TEASING CHILDREN.

BY UNCLE BOB.

This habit of teasing children is of so common occurence that it deserves attention. Some I hope will recognize the truth of what I say, and refrain hereafter from the cruel habit. That it is cruel is easily demonstrated. It is a mark of either intentional imposition or thoughtless negligence on the part of grown up people towards helpless little ones.

I know of persons who cannot be in the room with a child but a few minutes before they exhibit a desire to tease it. Some do it, imagining it is the way to play with them. If the child is an infant, they notice some marked desire which it has and tempt it to demand it. The little one's efforts and failures seem to please in the proportion in which anger is finally expressed.

It is impossible to enumerate the ways in which it is common to tease children. No reasoning person can fail to recall hundreds of cases. That they are trifling, I will admit. But by a long continued exercise of this teasing, the disposition of a child is rapidly soured. The child soon grows suspicious and learns to exhibit no desire in the presence of its tormentor, or else, it becomes bold and rude in its demands. Simplicity is a quality which makes, when combined with confidence, the loveliest trait in any little boy or girl.

It seems to me that no argument is needed to show the consequence of such early training. An injustice is perpetrated upon the helpless child. Its little strength is of no avail to redress its wrongs. The instinct of nature tells the sufferer that it ought to resent them, and that it cannot, occasions this result—a reaction upon the child's mind. What the subtle process is, we cannot explain. It is, however, analogous to the feelings in mature minds when suffering injury without power to redress or defend.

It is no wonder that some families possess a reputation for passion and revenge, when in the whole period of their lives no confidential or loving traits are cultivated.

It is common for nurses to exhibit the *spunk* of the little ones in their charge. Now a violent temper in a speechless infant is as bad as an equal exhibition in a full grown man. Helplessness and inability are the only measures of the danger to result from it.

A continuous cultivation of tender regard for individual rights will make a good tempered and easily trained child. The contrary treatment will produce a contrary disposition.

Blood and pedigree will not tell quicker nor as surely as will thoughtful treatment. Parents should see to it that no one is permitted to carelessly cross their children. They had much better have no company, than such poor company as entertains in this manner.

The older children should be taught early to recognize the rights of their younger brothers and sisters. Playmates should be checked when their plays have any show of imposition upon the weaker ones. It is a moral wrong, no matter whether young or old folks are concerned. Let my caution be heeded, and I know that many of the early associations of childhood will be pleasanter by reason of your care.

---

—"I say, cap'n," said a little-eyed man as he landed from the steamboat Petona, at Natchez, "I say, cap'n, this 'ere ain't all." "That's all the baggage you brought on board, sir," replied the captain. "Well, see, now, it's accordin' to list—four boxes, three chests, two ban' boxes, a portmanty, two hams (one part cut), three ropes, inyons, and a tea-kettle; but I'm dubersum. I feel there's something short, though I've counted 'em nine times, and never took my eyes off ov 'em while on board; there's something not right, somehow." "Well, stranger, the time's up; there's all I know of; so bring up your wife and five children out of the cabin, and we're off." "Them's um, darn it, them's um! I knowed I'd forgot something."

---

—An apothecary's boy was lately sent to leave at a house a box of pills, and at another six live fowls. Confused on the way, he left the pills where the fowls should have gone, and the fowls at the pills' place. The folks who received the fowls were astonished at reading the accompanying directions, "Swallow one every two hours."

---

—A chap was hung in Edinburgh, recently, upon whom was tried the experiment of producing instantaneous death by means of a 14 foot drop. It succeeded admirably, as his head was jerked off the first pop.

---

—According to the New York *Nation*, Professors Esmarch and Virchow have at last succeeded in introducing into the German field hospitals the American system of Barracks.

